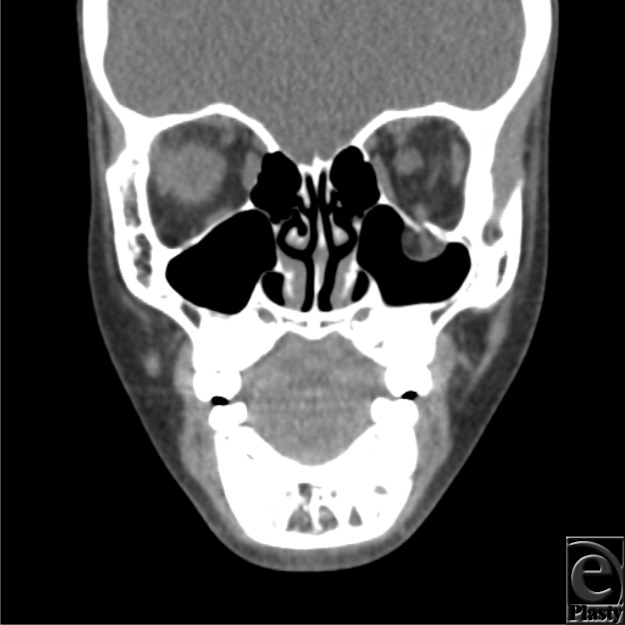# Pediatric Orbital Floor Fracture

**Published:** 2012-06-12

**Authors:** Mark E. Feldmann, Jennifer L. Rhodes

**Affiliations:** Division of Plastic and Reconstructive Surgery, Virginia Commonwealth University School of Medicine, Richmond, VA

## DESCRIPTION

A 7-year-old boy was brought to the emergency department with a chief complaint of vomiting multiple times over the previous 24 hours. He had been struck in the face by his elder brother's knee while wrestling the day prior.

## QUESTIONS

1. **What part of the orbit is most likely to fracture in a 2-year old? In a 7-year old? How does this correlate to changes in pediatric craniofacial anatomy?**
2. **How does a pediatric orbital floor fracture tend to present differently from the same injury in adults?**
3. **Describe the mechanism and symptoms of the oculocardiac reflex.**
4. **Is extraocular muscle entrapment more common in the pediatric or adult population? Why?**
5. **In the setting of suspected orbital floor fracture and diplopia, what is the optimal timing of repair in a pediatric patient? How does this differ from adults?**
6. **Describe special considerations for repairing pediatric orbital floor fractures.**

## DISCUSSION

Compared to adults, pediatric orbital floor injuries carry unique mechanistic features and fracture patterns. It follows that the prevalence, typical presenting signs and symptoms, and considerations for management of pediatric orbital floor fractures differ from adults as well.

Overall, the orbital floor is the most commonly fractured part of the orbit in a pediatric population, similar to adults. However, this injury is relatively uncommon in very young children, in whom most orbital fractures involve the roof rather than the orbital floor. This can be explained by anatomic changes. As a child grows, the midface enlarges relative to the forehead; the cheek fat pad atrophies, and the maxillary sinuses pneumatize, all leading to greater susceptibility of the floor to fracture. By age 7, orbital floor fractures surpass roof fractures in prevalence.^1^

Among all pediatric orbital floor fractures, most are “trapdoor” type (27%–93%).^2^ Trapdoor fractures are defined anatomically as linear, medially hinged, minimally displaced, and running along the infraorbital nerve canal. This “hinging” characteristic can allow recoil of the fractured bone, which in turn creates a scenario for transient herniation followed by entrapment of orbital contents within the fracture line. By contrast, “open door” fractures, which are much more prevalent in adults, are comminuted, creating a large orbital floor defect that allows herniation without entrapment.^3^ Differences in these fracture patterns are attributable to differences in bone characteristics between children and adults. Whereas the facial bones of children comprised elastic, cancellous bone with resilient periosteum, the orbital bone of adults has become mineralized and brittle, with fragile periosteum.^4^

Because of the difference in fracture characteristics between children and adults, disparities in presentation also exist. The adult orbital floor fracture is typically associated with periorbital edema and ecchymosis, subconjunctival hemorrhage, and diplopia. While children also tend to present with diplopia, ocular motility limitations are usually severe (44-100%),^2^ and there is frequently a notable absence of significant periorbital swelling or evidence of trauma to the globe. It is important to remember that diplopia in the setting of orbital floor fractures does not necessarily equate to entrapment of extraocular muscle. Other causes can include direct damage to the extraocular muscles during the injury, disruption of motor nerve branches, or, commonly, swelling and hemorrhage within the orbit causing limitation in globe excursion.

The unique clinical presentation of pediatric orbital floor fractures has been coined as “white-eyed blowout fracture,” marked by history of periocular trauma with equivocal or negative imaging findings, obvious extraocular muscle restriction, and absence of enopthalmos or periorbital soft tissue edema.^5^

Similarly, orbital injury may present with intractable nausea and vomiting, bradycardia, and occasionally syncope. This triad of symptoms forms the hallmark of the oculocardiac reflex, triggered by increased ocular pressure, and sensed by the ophthalmic division of the trigeminal nerve. Afferent signals traveling via the reticular formation to the motor nucleus of the vagus nerve result in parasympathetic stimulation of the heart and gut. In the setting of periocular trauma, the symptomatology of the oculocardiac reflex is highly suggestive of inferior rectus entrapment and can be considered an indication for surgery. Vomiting is the presenting complaint in approximately 1 of 4 children with entrapment^2^ and has a positive predictive value of 83.3% for entrapment when there is a known trapdoor type fracture.^6^

While imaging with ultrasound, MRI, and plain radiography for suspected orbital fracture have all been reported, CT scan is the modality of choice in a pediatric population. High-resolution, thin-cut, coronal acquisitions provide excellent imaging of the orbital bones. However, CT scan significantly underestimates extraocular muscle and soft tissue entrapment in children, with only 50% concordance between imaging and intraoperative findings, compared to 87% in adults.^7^ For this reason, strong clinical evidence of entrapment alone—even without radiologic confirmation—has been advocated as grounds for surgery in children, given the high incidence of a trapdoor injury pattern. Other indications for surgery include positive forced duction test, enopthalmos larger than 2 mm at presentation, and fractures larger than 1 cm.^2^

While there have been no prospective, randomized trials performed in children to determine optimal timing of orbital floor fracture repair, multiple studies show evidence that early repair (1-5 days, ideally <48 hours) results in improved long-term ocular motility over later intervention.^2^ In one study of 20 patients presenting with white-eyed blowout fracture, for example, those who underwent surgical repair within 24 to 48 hours had better long-term post-operative motility than those who underwent a standard 2-week waiting period before repair.^5^ Other accepted indications for immediate repair include diplopia with non-resolving oculocardiac reflex, and early enopthalmos or hypoglobus.

The approaches to repair of orbital floor fractures are similar between children and adults, including transconjunctival, transcutaneous, and transantral techniques in both populations. The transconjunctival approach has become the favored approach of most surgeons in children because of its avoidance of external scar and lower incidence of ectropion.

An array of autogenous, allograft, xenograft, and alloplastic reconstructive implants for orbital floors has been studied in adults, each with advantages and disadvantages. While the same choices are available for children with orbital floor fractures, a comparative analysis does not exist, and the choice of implant ultimately lies with the surgeon. The implants most often employed in children include porous polyethylene, autogenous bone, dura, silicone, fluorocarbon polymers, and gelatin film.^2^ In general, autogenous grafts, including calvarium, iliac, rib, and cartilage (nasal septum, ear, rib), have the advantage of lower rates of infection and foreign body reaction, as well as less potential for fibrosis, migration, and extrusion than most alloplastic implants. However, when using autogenous grafts in children, extra consideration must be given to the effect this has on the skeleton at the donor site and the attendant growth disturbances that may ensue. Alloplastic implants are durable, omit the need for a donor site, decrease operative time, can be shaped and contoured very specifically, and provide both absorbable and nonabsorbable options. In the patient presented, the authors used a poly-D,L-lactide acid flexible, absorbable plate (Resorb-X, KLS Martin, Jacksonville, FL), which is easily shaped and allows support of the orbital contents during fracture healing and callus formation.

The list of complications after orbital floor repair in children mirrors that of adults and includes infection, hemorrhage, ocular dysmotility, persistent diplopia, enopthalmos, infraorbital dysesthesia, implant-related problems, extra-ocular muscle or optic nerve injury/compression, poor cosmesis, and, very rarely, lacrimal injury, fistula formation, cyst, or mucocele. Persistent diplopia is by far the most common reported complication in children, with a greater than 50% incidence in children younger than 9 years. Often this resolves, with an average resolution time of 10 to 18 months.^8^ Reasons for the high incidence of persistent diplopia are speculative, possibly related to the trapdoor pattern of fracture resulting in direct damage to extraocular muscles, rapid callus deposition (<1 week) around the fracture, as well as more frequent failures and/or delays in diagnosis in children relative to adults.

In summary, pediatric orbital floor fractures are rare in very young children, becoming more frequent after the age of 7 years. Because the presentation of the injury is usually more subtle in children, a high index of suspicion is needed to make the diagnosis, particularly when diplopia accompanies a suggestive history of mechanism. Unlike adults, true trapdoor-type fractures and associated entrapment of extraocular muscle is the norm in the pediatric population. Symptoms of an oculocardiac reflex are highly suggestive of entrapment, even without accompanying radiographic evidence, and should prompt surgical intervention. Though no long-term prospective studies exist, the weight of the literature supports earlier intervention in children with dysmotility of the globe. Even with this approach, a period of persistent, usually self-limiting, diplopia can be expected postoperatively.

## Figures and Tables

**Figure:**
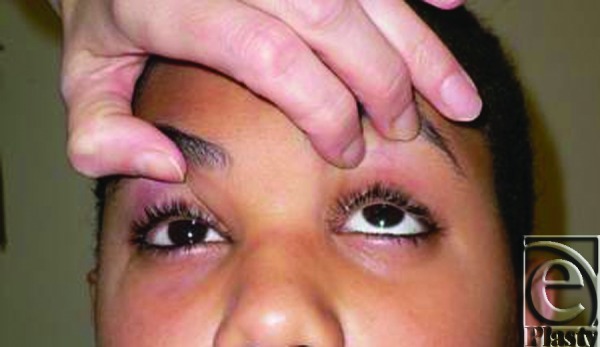


**Figure:**